# Rifaximin Versus Placebo for Irritable Bowel Syndrome: A Systematic Review and Meta-Analysis of Randomized Controlled Trials

**DOI:** 10.7759/cureus.116820

**Published:** 2026-09-24

**Authors:** Aymen A Alqurain, Aya Abdelrahman, Ibrahim H Alzahib, Zainab A Alsinan, Ayah A Edrissy, Asmaa O Alkhalil, Obaeda A Jamor, Yazeed A Alahmari, Omar A Almohmmadi, Abdulaziz A Alrehaili, Shahad O Alsubhi, Abdullah F Alrawashdeh, Ali A Alghamdi, Ali S Metwaly

**Affiliations:** 1 Clinical Practice, Northern Border University, Rafha, SAU; 2 Medicine, Cairo University, Giza, EGY; 3 Internal Medicine, King Salman Hospital, Tabuk, SAU; 4 Internal Medicine, Imam Abdulrahman Bin Faisal University, Qatif, SAU; 5 Medicine, Princess Nourah bint Abdulrahman University, Riyadh, SAU; 6 Internal Medicine, Almaarefa University, Riyadh, SAU; 7 Medicine and Surgery, Batterjee Medical College, Jeddah, SAU; 8 Medicine, King Khalid University, Khamis Mushayt, SAU; 9 Internal Medicine, King Fahd Hospital, Madinah, SAU; 10 Medicine, King Abdulaziz University, Jeddah, SAU; 11 Medicine, Universitatea de Medicină și Farmacie din Craiova, Craiova, ROU; 12 Medicine and Surgery, Umm Al-Qura University, Makkah, SAU; 13 Precision Medicine, Alexandria University, Alexandria, EGY

**Keywords:** dysbiosis, gut microbiota, irritable bowel syndrome, meta-analysis, rifaximin, systematic review, trial sequential analysis

## Abstract

Rifaximin, a minimally absorbed broad-spectrum oral antibiotic, is used to target gut dysbiosis in patients with irritable bowel syndrome (IBS). However, conflicting trial data and methodological heterogeneity necessitate an updated, rigorous synthesis of its efficacy and safety profile. We conducted a systematic review and meta-analysis of randomized controlled trials (RCTs) comparing rifaximin with placebo in adult patients with IBS, adhering to the Preferred Reporting Items for Systematic reviews and Meta-Analyses (PRISMA) 2020 guidelines. The primary outcome was global symptom relief at the end of the treatment. Data were pooled using inverse-variance random-effects models with Hartung-Knapp-Sidik-Jonkman (HKSJ) adjustment. Evidence robustness was evaluated using prediction intervals (PI), Trial Sequential Analysis (TSA), trim-and-fill algorithms for publication bias, and the Grading of Recommendations, Assessment, Development, and Evaluation (GRADE) methodology. Nine RCTs (yielding 10 randomized comparisons due to one trial's a priori stratification by small intestinal bacterial overgrowth status) were included in this review. For the primary outcome (eight comparisons, N=2,056), rifaximin significantly improved global symptom relief compared to placebo (RR 1.54, 95% CI 1.13 to 2.11; p=0.014), corresponding to a number needed to treat for benefit (NNTB) of 6. However, statistical heterogeneity was substantial (I^2^=59.5%), and the 95% PI crossed the line of no effect (0.89-2.67). The primary effect lost statistical significance upon the exclusion of high-risk-of-bias trials (RR 1.47, 95% CI 0.95-2.27) and after adjusting for publication bias using the trim-and-fill method (RR 1.37, 95% CI 0.93-2.01). The TSA indicated that the accrued information size remained inconclusive for a conservative 15% relative benefit. Rifaximin demonstrated no statistically detectable difference in adverse events compared to placebo, though this is accompanied by considerable uncertainty (Peto OR 0.89 (95% CI 0.65-1.22)). Rifaximin demonstrates a modest overall benefit for global IBS symptoms and an excellent safety profile; however, the certainty of evidence is very low. The apparent efficacy is highly fragile, driven largely by industry-funded trials, undermined by publication bias, and characterized by wide prediction intervals. Biomarker-guided stratification, such as testing for small intestinal bacterial overgrowth (SIBO), may be required to optimize patient selection in future independent trials.

## Introduction and background

Irritable bowel syndrome (IBS) is a prevalent functional gastrointestinal disorder estimated to affect 10%-15% of the global adult population [1]. Clinically characterized by chronic, recurrent abdominal pain associated with defecation or alterations in bowel habits, including diarrhea, constipation, or a mixed stool pattern, IBS imposes a substantial burden on healthcare systems [1-3]. This disorder is associated with significantly reduced health-related quality of life, increased work absenteeism, and impaired daily functioning [2,3]. Although the exact pathogenesis of IBS remains unclear, it is widely recognized as a multifactorial condition. Emerging evidence suggests an interplay between altered gastrointestinal motility, visceral hypersensitivity, low-grade mucosal inflammation, immune activation, and dysregulation of the gut-brain axis [4,5].

Recently, the role of intestinal dysbiosis, defined as qualitative and quantitative alterations in the gut microbiota, has been implicated as a driver of the pathophysiology of IBS, particularly in diarrhea-predominant (IBS-D) and mixed (IBS-M) subtypes [2,4]. A notable subset of patients with IBS exhibits small intestinal bacterial overgrowth (SIBO), linking microbial imbalance to heightened mucosal inflammation and symptom generation via bacterial fermentation [2,5]. Therapeutic strategies aimed at modulating the gut microbiota have gained significant attention. Conventional treatments, such as antispasmodics, tricyclic antidepressants, and dietary modifications, often yield suboptimal symptom relief and are burdened by long-term adherence challenges. Traditional systemic antibiotics carry significant risks, including systemic toxicity, opportunistic infections (e.g., *Clostridioides difficile*), and the rapid emergence of bacterial resistance [2,5,6].

Rifaximin, a semi-synthetic derivative of rifamycin, has emerged as a targeted therapeutic option to address these limitations. As a minimally absorbed broad-spectrum oral antibiotic, rifaximin exhibits negligible systemic bioavailability (<0.4%), allowing it to reach high concentrations within the gastrointestinal lumen [1,5]. Approved by the US Food and Drug Administration (FDA) for the treatment of IBS-D, rifaximin exerts its clinical efficacy through direct antimicrobial mechanisms, such as the eradication of SIBO and indirect eubiotic effects. These include stabilization of the gut microbiota, modulation of bacterial virulence, and reduction of localized mucosal inflammation and visceral hypersensitivity [4,6,7]. Extensive clinical evaluations, including the landmark TARGET 1, 2, and 3 randomized controlled trials (RCTs), have demonstrated that a short-course (two-week) regimen of rifaximin provides significant and sustained relief of global IBS symptoms, abdominal pain, and bloating [2,8]. Furthermore, both pairwise and network meta-analyses have highlighted its highly favorable benefit-to-harm ratio, ranking rifaximin as one of the safest pharmacological interventions for IBS, with adverse event profiles comparable to placebo and no clinically significant induction of bacterial resistance, even upon retreatment [2,3,6].

Despite the established therapeutic potential of rifaximin, the literature presents several inconsistencies. Variations in study populations, diagnostic criteria, treatment regimens, retreatment strategies, and follow-up durations have resulted in heterogeneous clinical findings across RCTs. Although previous meta-analyses have evaluated the utility of rifaximin, the recent publication of additional trials and the availability of extended follow-up data warrant an updated, rigorous, and comprehensive synthesis of the evidence [1,5,8]. Therefore, this systematic review and meta-analysis aimed to evaluate and synthesize data from RCTs comparing rifaximin with placebo in adults with IBS. By providing robust estimates of treatment efficacy regarding global symptom improvement, abdominal pain relief, bowel habit-related outcomes, quality of life, and safety, this study sought to elucidate the consistency of findings across trials and inform future clinical guidelines for the management of IBS.

## Review

Methods

Protocol and Registration

This systematic review and meta-analysis was conducted in accordance with the Preferred Reporting Items for Systematic Reviews and Meta-Analyses (PRISMA) 2020 guidelines [9]. The study protocol was prospectively registered in the International Prospective Register of Systematic Reviews (PROSPERO; CRD420261426893) and structured according to the PRISMA Protocols (PRISMA-P) statement [10].

Eligibility Criteria

Study eligibility was formulated using the Population, Intervention, Comparison, Outcomes, Study design (PICOS) framework. The population included adult patients (≥18 years) with a confirmed diagnosis of IBS based on any accepted symptom-based criteria (Manning, Rome I-IV) or physician confirmation. All subtypes (IBS-D, constipation-predominant [IBS-C], IBS-M, and unclassified) were eligible. The intervention was oral rifaximin therapy at any dosage or duration, encompassing both single-course and re-treatment regimens, and the comparison was placebo control matching the intervention.

The primary outcome was the proportion of patients who achieved global symptom relief at the end of the treatment period, while the secondary outcomes were global symptom relief at the end of follow-up, bloating relief, abdominal pain response, stool consistency response, health-related quality of life defined as the proportion achieving the IBS-QOL minimal clinically important difference (≥14 points), prevention of symptom recurrence, durable response, and safety (any treatment-emergent adverse event, serious adverse events, drug-related adverse events, withdrawals due to adverse events, and Clostridioides difficile infection).

Parallel-group RCTs were considered eligible. Cross-over randomized designs were also eligible a priori, with a pre-specified plan to extract first-period data only; no cross-over trial met the eligibility criteria, and all included comparisons were parallel groups. Observational studies, non-randomized and quasi-experimental trials, studies without a placebo comparator, trials combining rifaximin with another active agent without a distinct monotherapy arm, mechanistic or microbiota sub-studies reporting no eligible efficacy or safety outcomes, and mixed populations from which IBS data could not be extracted separately were excluded.

Information Sources and Search Strategy

A search was performed across MEDLINE, PubMed, Embase, Scopus, the Cochrane Central Register of Controlled Trials (CENTRAL), and Google Scholar from database inception to July 2026. No language or publication date restrictions were applied. The strategy utilized the Cochrane Highly Sensitive Search Strategy for identifying RCTs, combining controlled vocabulary (MeSH and Emtree) with free-text terms (e.g., "rifaximin", "Xifaxan", "irritable bowel syndrome", "IBS", "bloating") [11]. To minimize publication bias, gray literature and clinical trial registries (ClinicalTrials.gov and the WHO International Clinical Trials Registry Platform (ICTRP)) were systematically searched. Furthermore, conference proceedings from Digestive Disease Week (DDW), American College of Gastroenterology (ACG), and United European Gastroenterology Week (UEGW) were hand-searched to capture abstract-only data.

Backward and forward citation chasing of the included articles and relevant reviews was performed [12]. Search results were reported in compliance with the PRISMA Search (PRISMA-S) extension, and deduplication was conducted using the Bramer method [13].

Study Selection

Selection was performed in two stages (title/abstract and full-text screening) by two independent investigators following a pilot calibration exercise. Discrepancies were resolved by adjudication by a third reviewer. The inter-rater reliability of the full-text screening was assessed. Recognizing that the high prevalence of exclusions in systematic reviews triggers the kappa paradox, agreement was quantified using Gwet's AC1 as the pre-specified primary agreement statistic, reported alongside Cohen's κ and the prevalence-and-bias-adjusted κ (PABAK), with magnitudes interpreted using the Landis-Koch benchmarks [14]. The magnitudes of agreement were interpreted using the Landis and Koch benchmarks, incorporating the prevalence caveat [15]. The entire selection process is delineated in a PRISMA 2020 flowchart.

Data Extraction

Data were independently extracted by two reviewers using a piloted and structured data extraction form. The extracted variables included study characteristics, patient demographics, interventions (dose and duration), and pre-specified efficacy and safety endpoints. The standard errors of the mean (SEM) were converted to standard deviations by multiplying by √n for one trial reporting dispersion as SEM [11].

For clustered publications (e.g., secondary analyses, post hoc reports, and long-term follow-ups linked to parent trials), data were extracted once from the most comprehensive source to mitigate duplicate publication bias. When trial data were missing or ambiguous, the corresponding authors were contacted as pre-specified in the protocol.

Risk of Bias Assessment

The methodological quality of the included RCTs was independently appraised by two reviewers using the Cochrane Risk of Bias 2 (RoB 2) tool for parallel-group trials [16]. The studies were evaluated across five domains: randomization process, deviations from intended interventions, missing outcome data, measurement of the outcome, and selection of the reported result. Particular scrutiny was applied to domain three (handling of missing data, particularly regarding worst-case scenario imputations) and domain five (selective reporting in trials utilizing post hoc endpoint redefinitions).

Data Synthesis and Statistical Analysis

Meta-analyses were conducted when at least two methodologically comparable studies reported the same outcomes. Dichotomous outcomes were pooled as risk ratios with 95% confidence intervals using the inverse variance method, which was harmonized across every pooled analysis. Risk differences were computed in parallel to supply the absolute effects, and the I² obtained for each scale was reported separately, as the two can differ materially. Robustness to analytic choices was examined by refitting the primary model under alternative between-study variance estimators (Restricted Maximum Likelihood, Paule-Mandel, Sidik-Jonkman), under an unadjusted Wald interval, under a Mantel-Haenszel common-effect model, and with the odds ratio instead of the risk ratio.

Mean differences (MDs) were computed for continuous outcomes measured on uniform scales. When disparate instruments were used, the standardized mean difference (SMD) was pooled using Hedges' g, incorporating the small-sample correction factor J [17]. For rare safety events containing zero-event cells, the Mantel-Haenszel method without zero-cell continuity correction or the Peto odds ratio was utilized to avoid estimation bias [18].

Given the anticipated clinical and methodological heterogeneity across functional gastrointestinal disorder trials, a random-effects model was employed a priori as the primary pooling strategy. The between-study variance (τ²) was estimated using the DerSimonian-Laird method for binary outcomes and the Restricted Maximum Likelihood (REML) estimator for continuous outcomes [19]. The Hartung-Knapp-Sidik-Jonkman (HKSJ) method was chosen because it produces more conservative and accurate confidence intervals in sparse meta-analyses (k < 10), correcting the anti-conservative nature of standard Wald-type intervals that frequently lead to false-positive conclusions [20].

Heterogeneity and Subgroup Analyses

Statistical heterogeneity was evaluated using Cochran's Q test and quantified via the I² statistic with 95% CIs, along with absolute heterogeneity estimates (τ² and τ). Recognizing the limitations of I² in reflecting the dispersion of true effects, a 95% prediction interval (PI) was calculated for the primary outcome to estimate the anticipated range of true treatment effects in future clinical settings [21].

Pre-specified subgroup analyses examined IBS subtype, rifaximin dose, treatment duration, diagnostic criteria, duration of follow-up (<8 vs. ≥8 weeks), and publication era, using a random-effects test for subgroup differences with τ² estimated separately within strata. In adherence to established statistical guidelines requiring approximately 10 studies per covariate, meta-regression was omitted due to inadequate statistical power [11].

Sensitivity and Influence Analyses

The robustness of the primary findings was probed through a priori sensitivity and influence analyses. Leave-one-out meta-analysis, Baujat plots [22], and influence diagnostics comprising studentized residuals, DFFITS, Cook's distance, covariance ratios, leave-one-out τ² and leave-one-out Q, and DFBETAS were used to identify highly influential outlier studies [23]. Sensitivity models re-estimated the primary outcome after (1) excluding trials with a high overall risk of bias; (2) excluding data available only in abstract form; (3) excluding the trial contributing an IBS subgroup of a broader cohort on the grounds of indirectness; (4) excluding prospectively unregistered trials published in 2024; (5) excluding trials published before 2010; and (6) entering the two strata as a single comparison. Random-effects estimates were compared with the Mantel-Haenszel common-effect model. The exclusion of re-treatment trials was not required, and the primary analysis excluded them by design.

Publication Bias and Small-Study Effects

Small study effects were visually assessed using contour-enhanced funnel plots. Because traditional regression-based asymmetry tests (e.g., Egger's, Harbord's) lack statistical power and are highly prone to false positives when k < 10, they were deliberately omitted [11]. Instead, publication bias was quantitatively evaluated using the Doi plot and the Luis Furuya-Kanamori (LFK) index, a methodology specifically validated for meta-analyses with limited study counts [24]. Potential outcome-reporting bias was assessed using the Outcome Reporting Bias in Trials (ORBIT) framework [25]. Furthermore, considering the pervasive nature of pharmaceutical funding in pivotal IBS trials, the potential impact of industry sponsorship was evaluated critically.

Trial Sequential Analysis

A trial sequential analysis (TSA) was performed to determine whether the current evidence base was sufficiently powered and conclusive for the primary outcome. The required information size (RIS) was computed for two proportions from the pooled placebo event rate with α = 0.05 (two-sided) and 90% power, adjusted for heterogeneity using the diversity measure D², and monitoring boundaries were constructed with an O'Brien-Fleming α-spending function [26]. Because the RIS derived from the observed pooled effect is circular, the a priori scenarios - a 15% and a 20% relative benefit - are reported as the primary trial sequential analyses, and the observed-effect scenario is presented only as a sensitivity analysis.

Certainty of Evidence

The overall certainty of the evidence was evaluated for each outcome using the Grading of Recommendations Assessment, Development, and Evaluation (GRADE) approach [27]. The evidence was downgraded based on five domains: risk of bias, inconsistency, indirectness, imprecision, and publication bias. Imprecision was strictly judged against the Optimal Information Size (OIS) and the CI position relative to minimal important differences (MIDs; e.g., a ≥50-point change for IBS Symptom Severity Score (IBS-SSS) or ≥14-point change for IBS Quality of Life Instrument (IBS-QOL)), rather than solely relying on statistical significance (p < 0.05).

Software and Reproducibility

All statistical analyses were performed using R version 4.6.0 (R Foundation for Statistical Computing, Vienna, Austria) [28], utilizing the meta and metafor packages for synthesis, HKSJ adjustments, prediction intervals, and influence diagnostics [29,30].

Results

Study Selection and Patient Characteristics

The literature search yielded 1959 records (1938 from bibliographic databases and trial registers and 21 from forward and backward citation chasing). After removing 604 duplicates and 12 records for other pre-screening reasons, 1322 records underwent title and abstract screening. Of these, 1162 were excluded from the study. Full-text retrieval was sought for 181 reports; 172 were retrieved and assessed for eligibility criteria. A total of 164 reports were excluded, leaving eight reports detailing ten randomized comparisons across nine trials included in the final quantitative synthesis [31-38]. Inter-rater reliability during the screening phase was excellent (percent agreement = 0.968; Gwet's AC1 = 0.963, 95% CI 0.931 to 0.995). The study selection process is shown in Figure 1.

**Figure 1:**
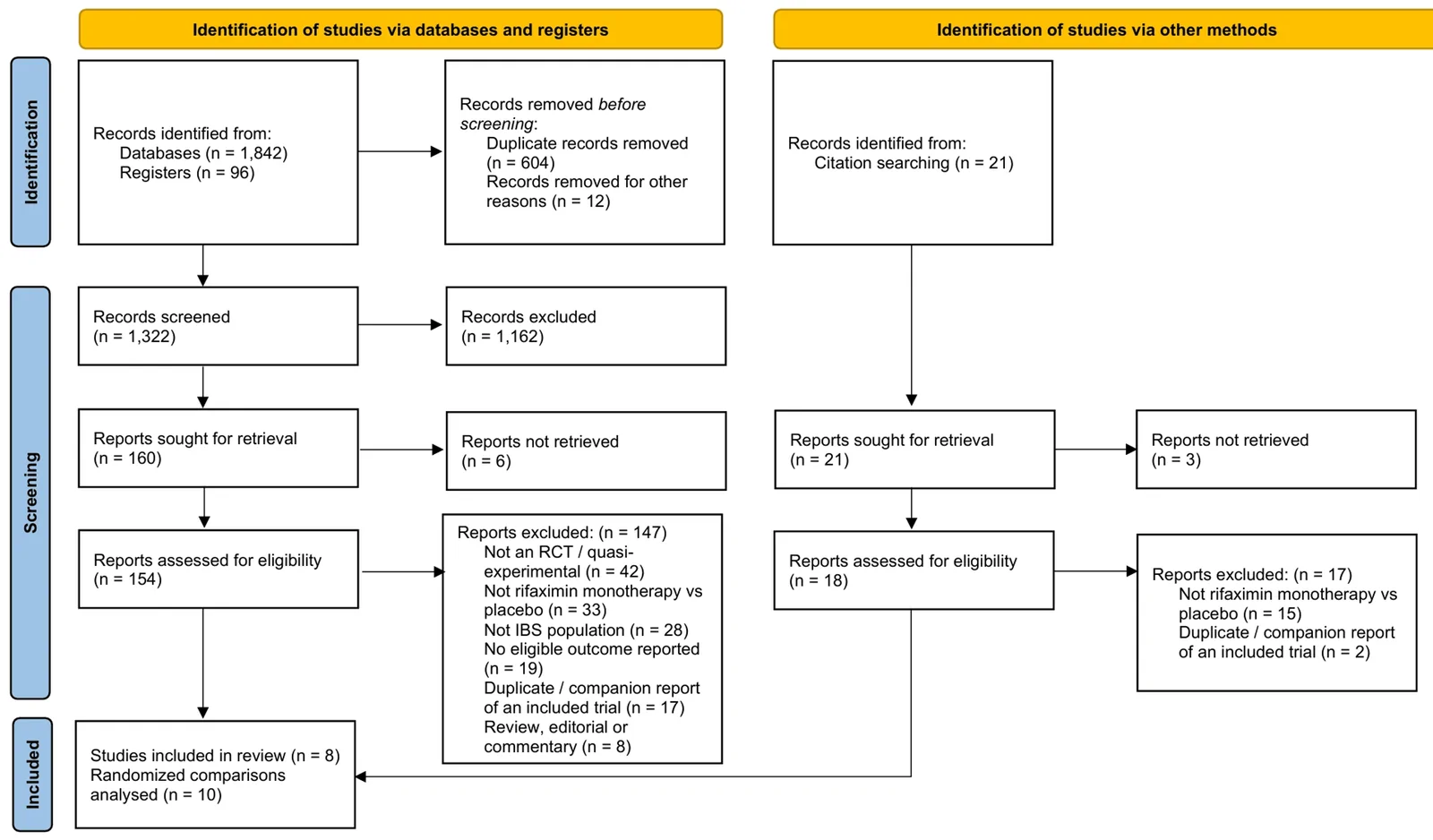
PRISMA 2020 flow diagram PRISMA - Preferred Reporting Items for Systematic Reviews and Meta-Analyses

The nine included trials randomized adult patients with IBS. The studies encompassed various diagnostic criteria (Rome I to IV) and IBS subtypes (IBS-D, non-constipated IBS, and mixed IBS). Rifaximin dosing ranged from 800 mg to 1650 mg/day, administered over 10 or 14 days, with follow-up durations extending from 1 to 16 weeks post-treatment. One trial was stratified into two independent comparisons based on the presence of small intestinal bacterial overgrowth (SIBO) [37]. Another trial (TARGET 3) utilized an open-label enrichment design followed by randomized re-treatment and was analyzed separately [36]. The trial characteristics are summarized in Table 1.

**Table 1:** Characteristics of the included trials and randomized comparisons ^a^ Evaluated a mixed Rome II IBS subgroup drawn from a broader functional bloating cohort; ^b^ Mechanistic trial reporting no eligible dichotomous outcome; dose not specified; ^c^ Utilized an open-label enrichment phase followed by randomized repeat treatment and analyzed separately from the single-course pool. Podder et al. [37] were pre-specified as two independent strata (small intestinal bacterial overgrowth positive and negative) with separate randomization lists and were therefore entered as two comparisons. IBS - irritable bowel syndrome; IBS-D - diarrhea-predominant IBS; SIBO - small intestinal bacterial overgrowth

Trial	Design & IBS subtype	Diagnostic criteria	Rifaximin dose (mg/day)	Treatment duration (days)	Assessment / follow-up (weeks)	N (rifaximin / placebo)	Overall risk of bias (RoB 2)
Pimentel et al., 2006 [31]	Single course, parallel (mixed)	Rome I	1200	10	10	43 / 44	Some concerns
Sharara et al., 2006 [32]	Single course, 3-phase parallel (mixed) ^a^	Rome II	800	10	1.4 (end of treatment); 2.9 (post-treatment)	37 / 33	High
Lembo et al., 2008 [33]	Single course, parallel (non-constipated)	Rome II	1100	14	12	191 / 197	Some concerns
TARGET 1 (Pimentel et al., 2011) [34]	Single course, parallel (non-constipated)	Rome II	1650	14	10	309 / 314	Low
TARGET 2 (Pimentel et al., 2011) [34]	Single course, parallel (non-constipated)	Rome II	1650	14	10	315 / 320	Low
Acosta et al., 2016 [35]	Single course, parallel (non-constipated) ^b^	Rome III	-	14	On-treatment	12 / 12	Some concerns
TARGET 3 (Lembo et al., 2016) [36]	Repeat treatment ^c^ (IBS-D)	Rome III	1650	14	4 (primary); 6 (durable response)	328 / 308	Some concerns
Podder et al., 2024 (SIBO+) [37]	Single course, stratified (non-constipated)	Rome IV	1500	14	4 (end of treatment); 16 (follow-up)	21 / 20	Some concerns
Podder et al., 2024 (SIBO-) [37]	Single course, stratified (non-constipated)	Rome IV	1500	14	4 (end of treatment); 16 (follow-up)	31 / 32	Some concerns
Haider et al., 2024 [38]	Single course, parallel (non-constipated)	Rome III	1200	14	1	75 / 74	High

Risk of Bias and Outcome Reporting Bias

The methodological quality varied across the included studies. Using the RoB 2 tool, TARGET 1 and TARGET 2 were assessed as having a low overall risk of bias [34]. Five trials demonstrated some concerns, driven by missing outcome data handling, deviations from the intended interventions, or lack of prospective trial registration [31,33,35-37]. Two trials were rated as having a high risk of bias due to selective reporting of an unplanned subgroup and unblinded per-protocol analyses, respectively [32,38]. Weighted by sample size, 46% of the evidence base was at a low risk of bias, 46% had some concerns, and 8% were at a high risk (Figures 2, 3).

**Figure 2:**
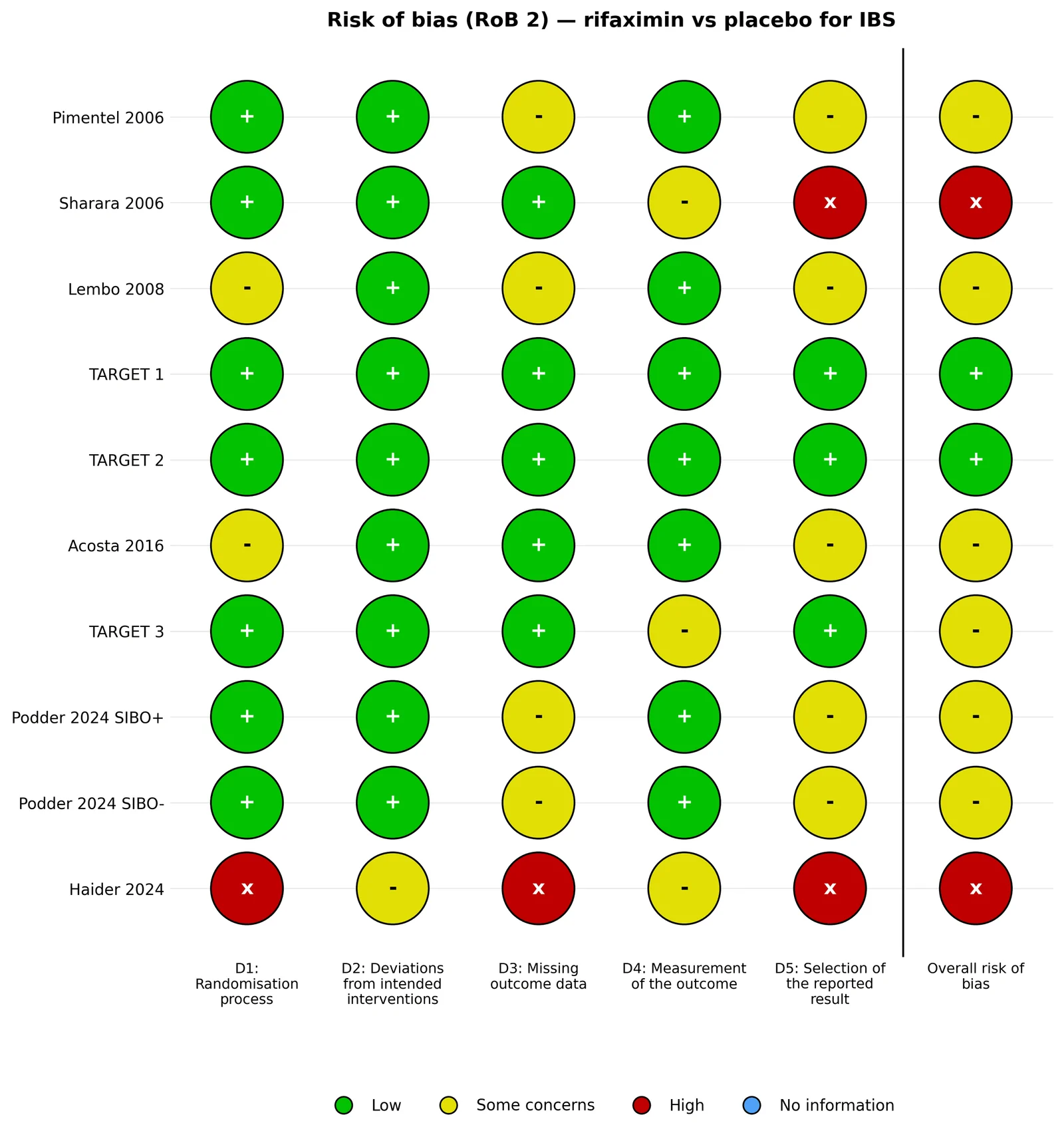
Risk of bias traffic plot Articles analyzed: [31-38]

**Figure 3:**
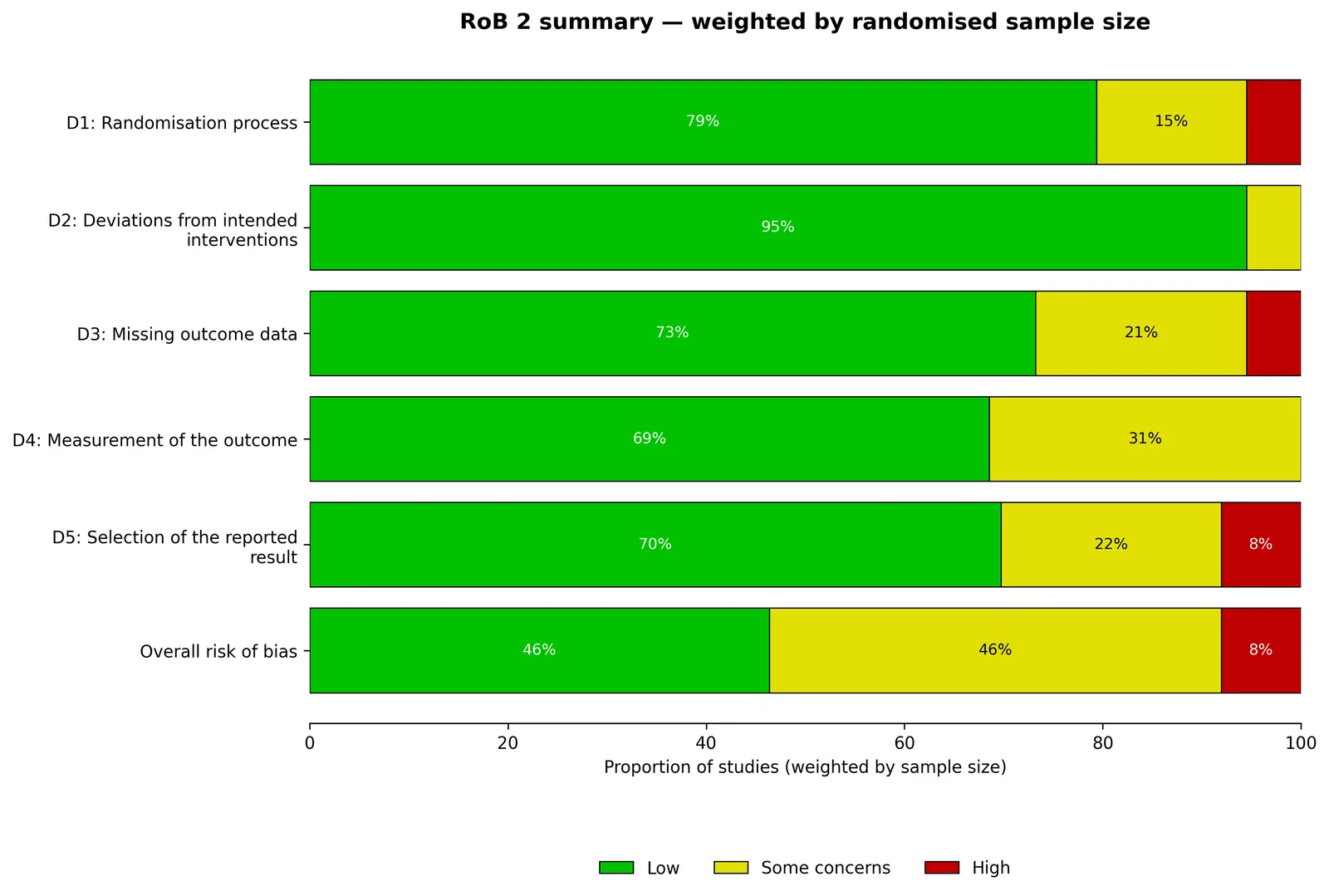
Risk of bias summary Weighted summary of risk-of-bias judgments across the included trials [31-38] using the Cochrane RoB 2 tool. Proportion of participants contributing to each risk-of-bias judgment within each domain is weighted by the number randomized. Overall, 46% of the evidence was rated at low risk, 46% had some concerns, and 8% was at high risk of bias.

Primary Outcome: Global Symptom Relief at End of Treatment

Eight single-course comparisons, encompassing 2056 participants (1022 randomized to rifaximin and 1034 to placebo), contributed to the primary efficacy outcome. Using a random-effects model with the HKSJ adjustment, patients treated with rifaximin were significantly more likely to achieve global symptom relief than those treated with placebo (pooled RR 1.54, 95% CI 1.13 to 2.11, p = 0.014). However, statistical heterogeneity was substantial (I^2^ = 59.5%, 95% CI 11.6% to 81.4%; τ² = 0.0422). The 95% PI ranged from 0.89 to 2.67, crossing the line of no effect, indicating that while the average effect favors rifaximin, the intervention may not be superior to placebo in all future clinical settings (Figure 4).

**Figure 4:**
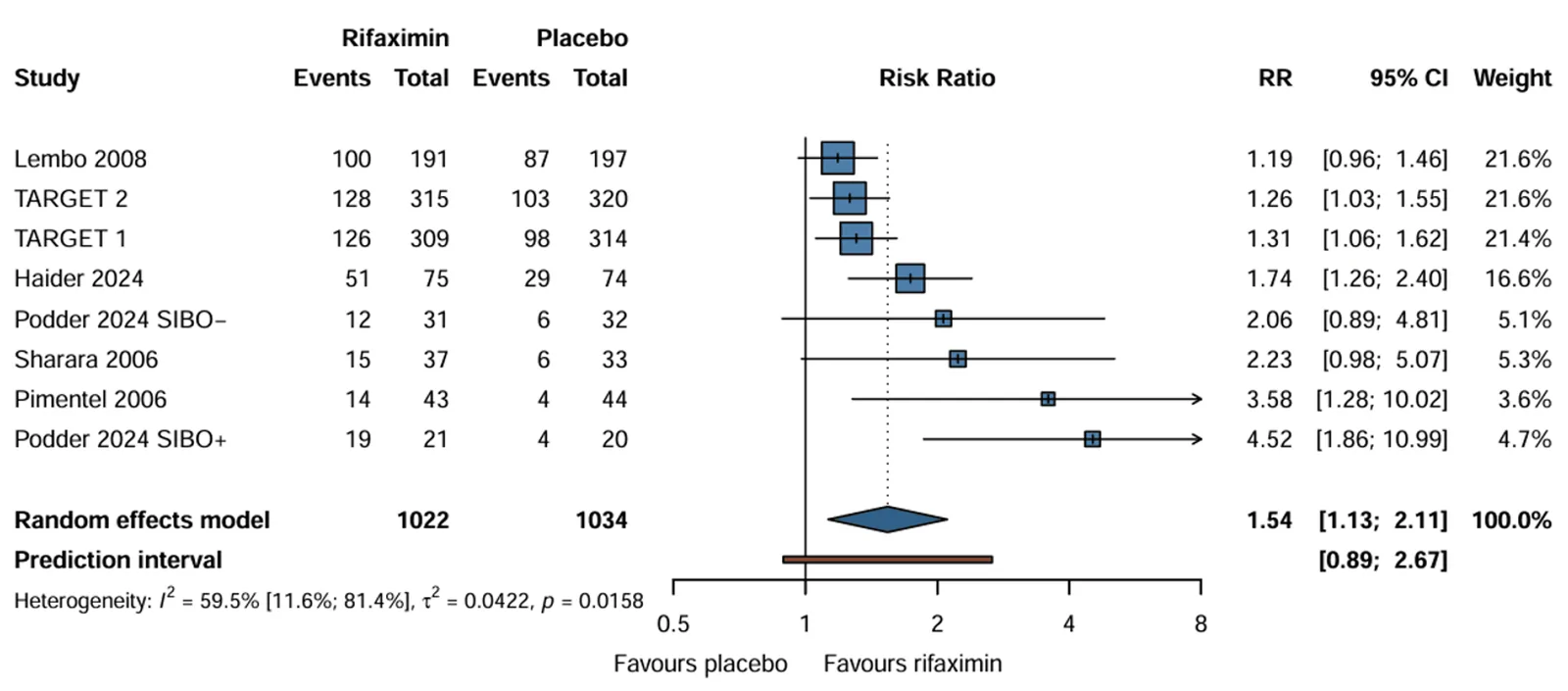
Forest plot for the primary outcome: global symptom relief at end of treatment Random-effects meta-analysis (DerSimonian-Laird estimator with Hartung-Knapp-Sidik-Jonkman adjustment) of eight single-course comparisons [31-34,37,38] evaluating the efficacy of rifaximin versus placebo for global symptom relief. Squares represent individual study risk ratios (RRs), with areas proportional to random-effects weights. Horizontal lines denote 95% confidence intervals. The diamond represents the pooled RR, and the horizontal bar beneath it signifies the 95% prediction interval (0.89 to 2.67), which crosses the line of no effect.

Based on the pooled placebo response rate of 32.6%, the absolute risk difference for global symptom relief was 0.176, corresponding to a number needed to treat for benefit (NNTB) of 6 (95% CI 3-25).

Subgroup, Sensitivity, and Influence Analyses

Pre-specified subgroup analyses exploring IBS subtype, treatment duration, total daily dose, diagnostic criteria, and publication era revealed no statistically significant subgroup differences that survived multiplicity considerations, serving as hypothesis-generating data.

Sensitivity analyses were performed to test the robustness of the primary outcomes. The pooled benefit lost statistical significance when the two trials at high risk of bias [32,38] were excluded (RR 1.47, 95% CI 0.95-2.27, p = 0.074). The estimate remained robust across various statistical estimators (REML, Paule-Mandel, Sidik-Jonkman) and upon exclusion of abstract-only data or pre-2010 trials. Influence diagnostics and Baujat plots identified TARGET 1, TARGET 2 [34], and the Podder et al. [37] SIBO-positive strata as contributing most significantly to overall heterogeneity. However, the leave-one-out meta-analysis confirmed that no single study completely drove the primary statistical significance; upon iterative removal of each trial, the pooled risk ratios remained relatively stable, ranging from a low of 1.41 to a high of 1.68.

Trial Sequential Analysis (TSA)

TSA was performed to determine whether the accrued evidence base is large enough to yield conclusive results. Assuming a conservative a priori 15% relative clinical benefit, the RIS adjusted for trial diversity was calculated to be 14,772 patients. The accrued sample size (N=2056) reaches only 14% of this requirement. Consequently, the cumulative Z-curve did not cross the O'Brien-Fleming α-spending monitoring boundary, indicating that the evidence base remains statistically inconclusive to confirm or reject a modest 15% relative benefit, highlighting the need for larger trials. However, when assuming a highly optimistic 20% relative benefit, the monitoring boundary was crossed.

Secondary Efficacy Outcomes

Four comparisons evaluated bloating relief at the end of treatment (N=1,733). The pooled effect favored rifaximin but failed to reach statistical significance (RR 1.31, 95% CI 0.98 to 1.76, p = 0.060; I^2^ = 32.1%). Similarly, global symptom relief evaluated at post-treatment follow-up (ranging from 3 to 16 weeks; k=4, N=562) favored rifaximin but was imprecise and not statistically significant (RR 1.85, 95% CI 0.72 to 4.73, p = 0.129; I^2^ = 56.3%).

Efficacy of Repeat Treatment (TARGET 3)

The TARGET 3 trial evaluated repeat rifaximin treatment in 636 patients with IBS-D who had previously responded to an initial open-label course but subsequently relapsed [36]. Due to its enrichment design, it was synthesized separately. At the end of the re-treatment period, the composite global relief did not differ significantly from placebo when utilizing the strict responder definition (RR 1.21, 95% CI 0.98 to 1.50). Similarly, bloating relief (RR 1.13, 95% CI 0.95-1.35) and stool consistency response (RR 1.04, 95% CI 0.89-1.21) showed no significant separation. However, re-treatment significantly improved the abdominal pain response (RR 1.20, 95% CI 1.01 to 1.42) and the proportion of patients achieving the IBS-QOL minimal clinically important difference (≥14 points) (RR 1.31, 95% CI 1.05 to 1.63).

Safety Outcomes and Absolute Effects

Rifaximin demonstrated a safety profile comparable to that of placebo across all measured parameters. Pooling via Peto odds ratios (to account for rare events and zero cells) revealed no significant difference between rifaximin and placebo for any adverse event (OR 0.89, 95% CI 0.65-1.22), serious adverse events (OR 0.94, 95% CI 0.23-3.78), or withdrawal due to adverse events (OR 0.75, 95% CI 0.32-1.79). Only a single case of C. difficile infection was reported in the rifaximin arm of TARGET 3 (occurring 37 days post-treatment following a course of cefdinir); the risk ratio was non-estimable, and confidence intervals included no difference. Consequently, the NNTH could not be estimated for any safety outcome due to the lack of risk divergence.

Publication Bias and Small-Study Effects

Contour-enhanced funnel plots were visually assessed (Figure 5), and small-study effects were quantitatively evaluated using the Doi plot and Luis Furuya-Kanamori (LFK) index. The LFK index was -0.05, indicating no major inherent asymmetries. However, applying the Duval and Tweedie trim-and-fill algorithm imputed three hypothetical missing studies on the left of the funnel. Adjusting for these imputed studies reduced the pooled risk ratio to 1.37 (95% CI 0.93 to 2.01, p = 0.103), effectively nullifying statistical significance. However, this adjustment must be interpreted as an exploratory sensitivity analysis for potential small-study effects rather than definitive confirmation of publication bias. The imputation of three missing studies contradicts the near-zero LFK index (-0.05, indicating symmetry), suggesting that the trim-and-fill algorithm's results may be a mathematical artifact driven by the small number of total comparisons (k=8) and underlying clinical heterogeneity.

**Figure 5:**
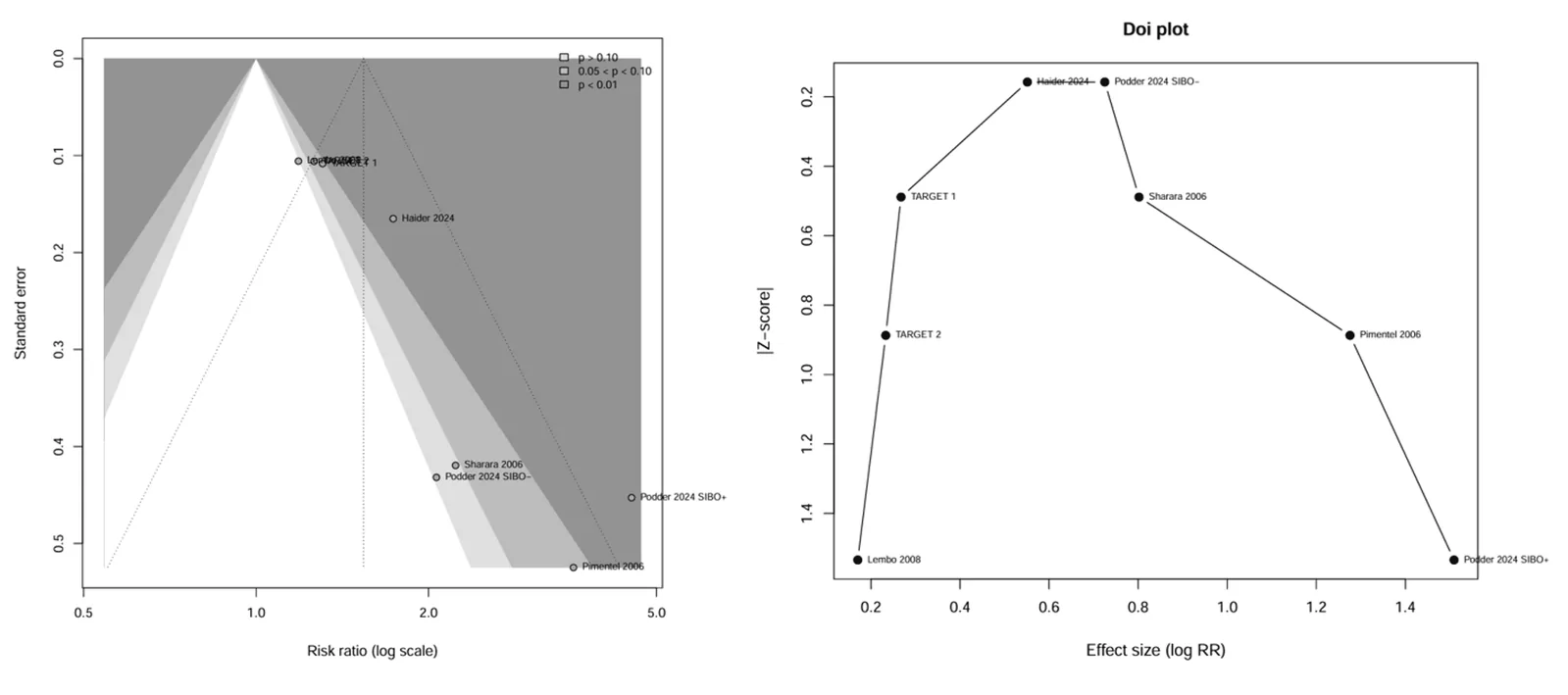
Contour-enhanced funnel plot and Doi plot for publication bias Assessment of small-study effects and publication bias for the primary outcome [31-34,37,38]. (A) Contour-enhanced funnel plot with shaded contours marking conventional significance thresholds; asymmetry suggests potential publication bias. (B) Doi plot displaying the absolute Z score against the effect estimate. The Luis Furuya-Kanamori (LFK) index of -0.05 indicates no major inherent asymmetry; however, trim-and-fill adjustments impute three missing studies, nullifying the pooled statistical significance.

Certainty of Evidence (GRADE)

The certainty of the evidence was evaluated using the GRADE framework (Table 2). The certainty of the primary outcome (global symptom relief) was rated as very low. It was downgraded by one level for risk of bias (significance lost upon removal of high-risk trials), one level for inconsistency (high I^2^ and a 95% PI crossing the line of no effect), one level for imprecision (wide HKSJ CIs), and one level for publication bias (near-universal industry funding and vulnerability to trim-and-fill adjustments). The evidence certainty for secondary endpoints, including bloating relief and safety outcomes, ranged from low to moderate, largely constrained by imprecision and indirectness due to trial enrichment strategies (e.g., TARGET 3). To ensure transparency regarding potential sponsorship bias, trial funding sources were systematically evaluated. Seven of the nine included trials, representing all studies contributing over 100 patients to the primary outcome (including the landmark TARGET trials), were funded by the manufacturer of rifaximin. While this indicates pervasive industry funding, evidence was not automatically downgraded for publication bias in the GRADE assessment on this basis alone unless corroborated by statistical asymmetry.

**Table 2:** GRADE summary of findings: rifaximin compared with placebo for irritable bowel syndrome GRADE summary of findings for the included trials [31-38]. Each domain column records the number of levels downgraded (0 = not downgraded; −1 = downgraded one level). Certainty starts at "High" for randomized controlled trials. The certainty in the primary outcome is judged as very low: the effect does not survive exclusion of trials at high risk of bias, heterogeneity is substantial with a prediction interval crossing the line of no effect, the statistical interval is wide, and trim-and-fill analysis nullifies the effect in the context of near-universal industry funding. CI - confidence interval; RR - risk ratio; OR - odds ratio; MCID - minimal clinically important difference; IBS-QOL - Irritable Bowel Syndrome Quality-Of-Life Instrument; HKSJ - Hartung-Knapp-Sidik-Jonkman; GRADE - Grading of Recommendations, Assessment, Development, and Evaluation

Outcome	No. of Comparisons (k)	Participants (N)	Pooled effect estimate (95% CI)	Certainty of evidence (GRADE)	Reasons for downgrading
Global symptom relief (end of treatment)	8	2056	RR 1.54 (1.13 to 2.11)	⨁◯◯◯ Very Low	Risk of Bias (-1): Significance is lost when high-risk trials are excluded. Inconsistency (-1): I2 = 60%; prediction interval crosses 1. Imprecision (-1): Wide HKSJ interval. Publication Bias (-1): Near-universal industry funding; trim-and-fill nullifies the effect.
Bloating relief (end of treatment)	4	1733	RR 1.31 (0.98 to 1.76)	⨁⨁⨁◯ Moderate	Imprecision (-1): The confidence interval includes no effect.
Global relief at follow-up	4	562	RR 1.87 (0.73 to 4.81)	⨁⨁◯◯ Low	Risk of Bias (-1): 2 of 4 trials unregistered. Inconsistency (-1): I2 = 59%. Imprecision (-1): Wide confidence interval (0.73 to 4.81).
IBS-QOL responder (MCID ≥ 14 points)	1	636	RR 1.31 (1.05 to 1.63)	⨁⨁◯◯ Low	Indirectness (-1): Enrichment population of prior responders (TARGET 3). Imprecision (-1): Derived from a single trial.
Any adverse event	3	1932	Peto OR 0.89 (0.65 to 1.22)	⨁⨁⨁◯ Moderate	Publication Bias (-1): Aggregate data relies solely on the manufacturer's pooled analysis.
Serious adverse events	1	636	Peto OR 0.94 (0.23 to 3.78)	⨁⨁⨁◯ Moderate	Imprecision (-1): Highly sparse data (8 events in total).
Withdrawal due to adverse events	4	1983	Peto OR 0.76 (0.32 to 1.79)	⨁⨁⨁◯ Moderate	Imprecision (-1): Highly sparse data (21 events in total).

Discussion

This updated systematic review and meta-analysis of RCTs evaluated the efficacy and safety of rifaximin in the management of IBS. While our primary analysis yielded a statistically significant pooled risk ratio (RR 1.54) favoring rifaximin for global symptom relief at the end of treatment, sensitivity and bias-adjustment analyses revealed a highly fragile evidence base. The apparent absolute risk reduction translates to an appealing NNTB of 6; however, the certainty of this evidence was downgraded to very low under the GRADE framework. The statistical significance of the primary outcome was abolished upon exclusion of trials at high risk of bias and following trim-and-fill adjustments for publication bias. Furthermore, TSA demonstrated that the current accrued sample size was underpowered to confirm a conservative 15% relative benefit. Conversely, the safety profile of rifaximin was unequivocally favorable, with incidence rates of overall and serious adverse events indistinguishable from those of placebo.

Our findings stand in stark contrast to the equivocal conclusions of earlier meta-analyses [1,3,5]. Previous syntheses, including the landmark network meta-analysis by Black et al. [3] and pairwise evaluations by Menees et al. [1] and Li et al. [5], endorsed rifaximin as a highly efficacious and safe intervention for IBS-D. The divergence of our conclusions from this prior consensus stems from our application of advanced, highly conservative meta-analytic methodologies designed to mitigate the risk of false-positive conclusions in sparse datasets [20]. By employing the HKSJ adjustment, we corrected the anti-conservative nature of the standard Wald-type confidence intervals utilized in older reviews. Additionally, while previous reviews relied on standard funnel plots, which lack statistical power for small study counts (k < 10), we implemented contour-enhanced funnel plots, the LFK index [24], and Duval and Tweedie's trim-and-fill algorithm. These rigorous diagnostic tools unmasked a pervasive small-study effect and publication bias that, when mathematically imputed, nullified the clinical efficacy of rifaximin in patients with IBS-D.

A critical addition to our analysis was the calculation of a 95% PI. While the 95% CI estimates the average treatment effect across the included trials, the 95% PI (0.89 to 2.67) estimates the expected true treatment effect in a future clinical setting [21]. The fact that the lower bound of the PI crosses the line of no effect indicates that, despite a positive average effect, rifaximin may not be superior to placebo in all clinical scenarios or patient subgroups.

This substantial heterogeneity (I^2^ = 59.5%) was driven by variations in the baseline microbial pathophysiology among the enrolled cohorts. For instance, a recent trial by Podder et al. [37] demonstrated a profoundly magnified treatment effect in patients prospectively stratified for small intestinal bacterial overgrowth (SIBO-positive RR 4.52 vs. SIBO-negative RR 2.06), aligning with the mechanistic rationale that rifaximin's primary therapeutic mechanism in IBS is the eradication of bacterial overgrowth and modulation of microbial fermentation [2,7]. The indiscriminate prescription of rifaximin to unstratified IBS populations, particularly in the absence of objective breath testing, may dilute the drug's apparent efficacy and drive the heterogeneity observed in our pooled estimates.

Furthermore, the results of the TARGET 3 re-treatment trial [36] highlight the complexities of long-term IBS management. Because TARGET 3 utilized an open-label enrichment design, randomizing only those who successfully responded to the initial course of rifaximin, the cohort represents a highly selected population. Even within this enriched cohort, re-treatment failed to show statistically significant superiority over placebo for composite global relief (RR 1.21, 95% CI 0.98 to 1.50) or bloating, achieving significance only in secondary measures such as abdominal pain and quality of life.

The strengths of this systematic review are its methodological rigor and exhaustive interrogation of bias. The a priori protocol registration, strict adherence to the PRISMA 2020 guidelines [9], and the use of robust statistical techniques (HKSJ adjustments, prediction intervals, TSA, and LFK indices) ensured a highly conservative and clinically realistic appraisal of the data.

Nevertheless, several limitations of this study must be acknowledged. First, the lack of patient-level data precludes conducting a definitive meta-regression to identify patient-specific covariates (e.g., baseline microbiome diversity, precise IBS subtype, or transit times) that dictate treatment response. Second, the secondary outcomes, particularly global relief at follow-up, suffered from significant data sparsity and reporting inconsistency across trials, resulting in wide CIs and limiting conclusions regarding the durability of rifaximin effects. Finally, the evidence base is compromised by industry-sponsored bias. Seven of the nine included trials, representing all studies contributing over 100 patients to the primary outcome, were funded by the manufacturer of rifaximin [34,36]. As established in the literature, pervasive industry funding correlates with outcome reporting bias, which is strongly corroborated by our trim-and-fill analysis [25].

## Conclusions

Rifaximin provides modest, short-term therapeutic benefits for global IBS symptoms, with an adverse event profile indistinguishable from that of placebo. However, the foundational evidence supporting its broad efficacy is fragile, heavily reliant on industry-sponsored trials, and sensitive to publication bias and methodological quality. Given that the 95% PI suggests that rifaximin will not be effective in all future clinical settings, indiscriminate use in unselected IBS populations may yield suboptimal outcomes. Future research must pivot away from broad all-comer IBS trials. Instead, rigorously designed, independent (non-industry-funded) RCTs should focus on biomarker-driven patient selection, specifically identifying the clinical utility of rifaximin in subgroups with confirmed SIBO or well-characterized dysbiosis. Until such data emerge, the prescription of rifaximin should be individualized, weighing the high safety profile against the very low certainty of consistent therapeutic benefits.
